# Supplementary material for: Low prevalence of Plasmodium falciparum histidine-rich protein 2 and 3 gene deletions in malaria-endemic states of India
Source: PLoS One. 2024 Dec 18;19(12):e0315662. doi: 10.1371/journal.pone.0315662 (PMC11654920; doi:10.1371/journal.pone.0315662)
Supplement: Attachment — Submitted filename: Response.docx [file pone.0315662.s001.docx]

Dear Editors,

Thank you very much for the opportunity to correct the mistakes in our manuscript.

In response to your queries, we have addressed all the issues in the current version of the manuscript.

**Q1:** In your manuscript you have stated that: 'The details regarding primers and PCR cycling parameters employed for the amplification of Pfhrp2 and Pfhrp3 are described in Table 1.' However, Table 1 instead contains the distribution of hrp2/hrp3 deletion cases across Indian states/year.

**Response:** We have corrected Table 1, which presents the details of the primers used in the study.

**Q2:** Please note that for manuscripts to be considered for publication in PLOS ONE, experiments and analyses must be conducted rigorously, with appropriate controls; methods must be described in sufficient detail for others to replicate the analyses; and conclusions must be supported by the results presented (http://journals.plos.org/plosone/s/criteria-for-publication). Therefore, could you kindly revise your manuscript to upload the correct Table 1 described in your methods.

**Response:** We have expanded the methodology section in greater detail. Please refer to lines 93-98 for the updated information.

**Q3:** Please also consider whether you need to update your methodology to provide sufficient detail for others to replicate your experiments for example, by providing further details of the statistical analyses done.

**Response:** We have included a data analysis section in the manuscript. Please refer to lines 99-101 for the details.
